# Training Robust T1-Weighted Magnetic Resonance Imaging Liver Segmentation Models Using Ensembles of Datasets with Different Contrast Protocols and Liver Disease Etiologies

**DOI:** 10.21203/rs.3.rs-4259791/v1

**Published:** 2024-04-30

**Authors:** Nihil Patel, Mohamed Eltaher, Rachel Glenn, Kari Brewer Savannah, Kristy Brock, Jessica Sanchez, Tiffany Calderone, Darrel Cleere, Ahmed Elsaiey, Matthew Cagley, Nakul Gupta, David Victor, Laura Beretta, Adrian Celaya, Eugene Koay, Tucker Netherton, David Fuentes

**Affiliations:** The University of Texas MD Anderson Cancer Center; The University of Texas MD Anderson Cancer Center; The University of Texas MD Anderson Cancer Center; The University of Texas MD Anderson Cancer Center; The University of Texas MD Anderson Cancer Center; The University of Texas MD Anderson Cancer Center; The University of Texas MD Anderson Cancer Center; Houston Methodist; Houston Methodist; The University of Texas MD Anderson Cancer Center; Houston Methodist; Houston Methodist; The University of Texas MD Anderson Cancer Center; The University of Texas MD Anderson Cancer Center; The University of Texas MD Anderson Cancer Center; The University of Texas MD Anderson Cancer Center; The University of Texas MD Anderson Cancer Center

**Keywords:** liver segmentation, T1 MRI, deep learning, robustness, multi-dataset training

## Abstract

Image segmentation of the liver is an important step in several treatments for liver cancer. However, manual segmentation at a large scale is not practical, leading to increasing reliance on deep learning models to automatically segment the liver. This manuscript develops a deep learning model to segment the liver on T1w MR images. We sought to determine the best architecture by training, validating, and testing three different deep learning architectures using a total of 819 T1w MR images gathered from six different datasets, both publicly and internally available. Our experiments compared each architecture’s testing performance when trained on data from the same dataset via 5-fold cross validation to its testing performance when trained on all other datasets. Models trained using nnUNet achieved mean Dice-Sorensen similarity coefficients > 90% when tested on each of the six datasets individually. The performance of these models suggests that an nnUNet liver segmentation model trained on a large and diverse collection of T1w MR images would be robust to potential changes in contrast protocol and disease etiology.

## Introduction

Liver cancer ranks among the leading causes of cancer deaths worldwide, accounting annually for more than 700,000 deaths^1^. It is considered a major health problem in the United States and globally. Several treatment strategies, such as surgical resection and Y-90 radioembolization, require accurate volumetric analysis of the liver and tumor(s) which is often achieved by segmenting the liver and the accompanying lesions on medical images, such as computed tomography (CT) and magnetic resonance imaging (MRI). Although the gold-standard method of segmentation is manual delineation by a trained radiologist, this method is very time-consuming, less reproducible, and prone to inter- and intra-observer variability.

In recent years, deep learning models have been trained to perform automated liver segmentation as an alternative to manual delineation. Jansen et al used a fully convolutional network as part of a liver metastasis detection pipeline to achieve a 95% Dice-Sorensen coefficient (DSC) when trained on 55 DCE-MRI series^2^. Isensee et al submitted a self-configuring nnUNet framework to both the LiTS and CHAOS challenges and finished first in both challenges, scoring mean DSCs of 95% on 131 CT series in the LiTS challenge and 75% on 60 MRI series in the CHAOS challenge^3–5^. Bibars et al used the CTs in both the LiTS and CHAOS datasets to pretrain the encoder of a 2-D U-Net and then fine-tuned the decoder on MRIs from the Duke Liver Dataset (DLDS), achieving a mean DSC of 88%^4–7^. Lambert et al trained anisotropic hybrid U-Nets (AHUNets) with 2D encoders and 3D decoders on the ATLAS dataset on the task of segmenting both the liver and the tumor^8,9^, achieving a mean DSC of 94%, mean Hausdorff distance of 2.85 mm, and mean surface DSC of 81% on the liver segmentation task. Hossain et al trained a 2D cascaded network on all 40 T1-weighted MRI series in the CHAOS dataset using 5-fold cross-validation and data augmentation and achieved a mean validation DSC of 95.15% when segmenting the liver^10^. Due to the relatively small size of publicly-available MRI datasets, it’s not uncommon for researchers to use larger amounts of institutional data. Kart et al trained nnUNet on a dataset comprising 400 T1-weighted MR images and achieved a mean DSC of 98% on a liver subtask of abdominal organ segmentation^11^. However, the images were obtained from healthy individuals, which may limit the model’s generalizability when confronted with MRI from liver cancer patients, in whom the liver contour is often deformed.

Because liver tumors have different etiological factors and morphologies^12^, its effects on the shape, boundaries, and volume of the liver can vary significantly. A model that is robust to these changes, therefore, must be trained on imaging data from as many unique patients with as many different etiologies as possible. Recently, Wasserthal et al unveiled TotalSegmentator, a single nnUNet model trained on CT images from 1204 patients, 655 of which had six different pathologic diagnoses, each with 104 labeled anatomical structures; TotalSegmentator achieved a mean DSC of 96% when tested on a liver CT segmentation sub-task of the Beyond the Cranial Vault Challenge^13,14^.

Motivated by the success of TotalSegmentator, the current paper develops a robust deep learning model for segmenting T1-weighted liver MRI using a large number of diverse datasets. As such, our goal was to curate a large heterogenous dataset consisting of MR images from a variety of patients and healthy subjects obtained from both publicly available and local datasets (from our institution). Our hypothesis is that the variation and diversity in imaging sequences, artifacts, and contrast agents’ protocols seen across the dataset would allow us to train a robust deep learning model. To this effect, we conducted two experiments using three different deep learning architectures: SMIT, nnUNet, and the Medical Imaging Segmentation Toolkit (MIST), henceforth referred to as PocketNet^3,15,16^.

Our experiments resulted in multiple deep learning models each trained on different datasets with different sequences, contrast agent types, artifacts, and etiologies, making these models as robust as the availability of curated data allowed for.

## Materials and Methods

### Datasets

Inclusion criteria for MR images used for training, validation, and testing were as follows: 1) entire liver visible in the image; 2) presence of all eight liver segments, i.e., no history of hepatectomy or lobectomy prior to image acquisition; 3) acceptable image quality such that the boundary liver is identifiable without the use of a pre-existing contour. We manually inspected and determined whether any identified MR images met these criteria using relevant metadata regarding the patients and/or datasets that the scans were obtained from, in addition to examination of the MR images. The primary features used to identify the liver segments included the presence or absence of the left and right portal veins along with tissue homogeneity. Images from patients who had undergone hepatectomy or lobectomy were excluded. When we used multiple MR images from the same patient, we ensured that these MR images were not split across the training, validation, and testing sets of our models. Finally, we used MR images with N4 bias field correction applied^17^.

We collected a total of 819 T1-weighted MR images from a total of 312 patients. Of these, 72 patients had cirrhosis, a risk factor and common finding in patients with primary hepatocellular carcinoma, who underwent MRI at the Duke Cancer Institute (data collected from the Duke Liver Dataset [DLDS])^6^. Another 34 patients with liver cancer underwent MRI at The University of Texas MD Anderson Cancer Center. An additional 71 patients with hepatocellular carcinoma who underwent MRI were collected from Houston Methodist Hospital. Fifty-eight anonymized patients with hepatocellular carcinoma who underwent MRI at the Bourgogne University in Dijon were also included (data collected from A Tumor and Liver Automatic Segmentation [ATLAS] dataset)^9^. Also, 57 patients with “abdominal tumors/abnormalities” underwent MRI at the Longgang District People’s Hospital in China with a protocol approved by the hospital’s Research Ethics Committee (data collected from the Abdominal Multi-Organ Segmentation [AMOS] dataset)^18^. Although a small subset of these patients’ scans showed tumor growth and lesions on the liver itself, most patients had unrelated abnormalities. Finally, 20 healthy individuals underwent MRI at the Dokuz Eylul University Hospital’s Department of Radiology in Izmir, Turkey, using an Institutional Review Board–approved protocol (data collected from the Combined Healthy Abdominal Organ Segmentation [CHAOS] dataset)^5^.

**Figure 1:**
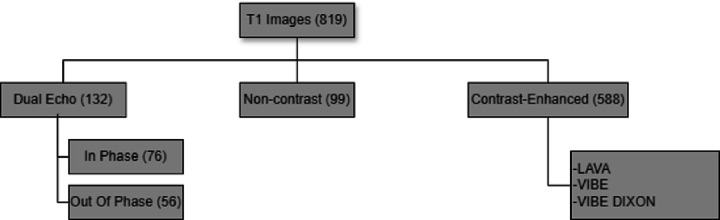
Contrast type breakdown of T1-weighted magnetic resonance images used in our study

Ranges of repetition times, echo times, and contrast agents of the public datasets used are provided in Table 1. A further breakdown of all datasets we used is provided in Fig. 1 and Table 2.

### Model Descriptions

Our experiments involved training three different liver segmentation models: SMIT, PocketNet, and nnUNet.

#### SMIT

Although we refer to this model as a “SMIT model,” SMIT itself is a student-teacher pretraining setup with the goal of training a sliding window (Swin) transformer encoder to accurately partition and encode medical images for inference purposes.^15^ The pretrained encoder is then used in a fully convolutional network that is then fine-tuned on a different task such as segmentation or classification. Because the encoder uses a Swin Transformer, input images are broken up into patches of a fixed size for both training and inference.^19^

Preprocessing is done as part of training, validating, and testing the model. The MR images are re-oriented to the RAS + orientation and resampled to a uniform voxel spacing of 1.5 mm × 1.5 mm × 2.0 mm^15^. Each image is then Z-score–normalized using its mean and standard deviation. If the image is smaller than the region of interest, it is spatially padded in whatever dimension is smaller than that of the patch.

After this, different steps occur depending on whether the MR images are used for training. Training MR images are randomly cropped into 4 patches of 96 × 96 × 96 voxels^15^. Finally, data and intensity augmentation are performed on the training MR images. These steps include randomly rotating the image 90 degrees along the x and y planes up to 45 times, randomly scaling the voxel intensities by a factor of 1.1, and randomly shifting the intensity distribution by an offset of 0.1. By default, the rotation has a 20% chance of occurring, and the intensity scaling and shifting has a 10% chance of occurring. These steps are not applied to validation or testing MR images.

The original preprocessing code written by the creators of the SMIT model did not perform Z-scoring, instead rescaling the MR image intensity to a range of [−175,250]^15^. However, because the model would be trained on multiple different sequences gathered from different institutions, we theorized that Z-scoring would allow us to introduce more uniformity to the data. Preliminary experiments in training our own SMIT models seemed to prove our theory correct, because the change resulted in an improvement in both validation and testing DSCs compared with the original scheme.

We used the pretrained encoder weights provided by the SMIT creators for fine-tuning. We also added a SoftMax activation function after the final convolution layer. Originally, SoftMax was applied only to the logits during training loss computation. Training loss is computed as a weighted sum of Dice loss and cross-entropy loss.

All SMIT models were fine-tuned for a maximum of 2,000 epochs and were evaluated every 10 epochs. We used AdamW^20^ for parameter optimization with an initial learning rate of 2E-5, which we chose based on a parameter search. The learning rate is increased linearly for a warmup period of 50 epochs and then annealed using cosine scheduling.

Because SMIT is built upon the SWin-UNetR architecture^21^, it performs inference using a sliding window with a patch size of 96 × 96 × 96 voxels. Window overlap was set to 50%, and Gaussian blending was applied to the prediction windows with a standard deviation scale of 0.125.

#### PocketNet

The PocketNet model aimed to reduce the number of parameters, and thus the computational power, needed to train convolutional neural networks on medical image analysis tasks without compromising performance^16^. Unlike most CNN architectures, PocketNet uses a constant amount of feature maps for each convolutional layer, drawing upon a paradigm that suggests that the traditional doubling of feature maps is unnecessary due to similarities between the features of U-Net-like architectures and those of classical multigrid algorithms that use a hierarchy of independent grids to decompose an image into different resolutions, thereby maintaining any information that might be lost during downsampling.^16^

A PocketNet implementation of nnUNet, with 32 feature maps in each convolution layer, was used for this project. Preprocessing steps included first reorienting the MR images to the right-anterior-inferior direction and resampling them to the mean spacing of the training data. Next, the intensities were windowed to the 0.5–99.5 percentile, followed by Z-score normalization. Finally, data augmentation was performed using random ratios, random intensity scaling, random elastic deformations, gamma corrections, and mirroring.

Both training and inference used a sliding window with a patch size of 256 × 128 × 128 voxels of the cropped patches used for training and a stride set to half the patch size. Prediction patches were blended back together using Gaussian blending with a standard deviation scale of 0.125. The models were trained for a total of 200 epochs using the ADAM optimizer with an initial learning rate of 3E-4 and the number of steps per training epoch set to the length of the model’s training set.

#### nnUNet

Since its introduction, nnUNet has become a popular tool for use in medical image segmentation because its ability to automatically configure a preprocessing and deep learning training pipeline based on the properties of its training data eases the burden of manually developing models to suit a particular data modality^3^.

We chose specifically to train 3D full-resolution U-Nets using nnUNet as a baseline for comparison against the other two models.

The default settings for experiment planning, preprocessing, model training, and inference were used in all experiments^3^. Postprocessing of inference outputs consisted of keeping the largest connected component.

## Experiments

Using the data we curated and the three models described in the prior sections, we conducted two experiments to evaluate each model’s performance on MRI liver segmentation both when trained on a single dataset and when trained on ensembles of datasets.

### Experiment 1: One-Source 5-Fold Cross-Validation

**Figure 2.**
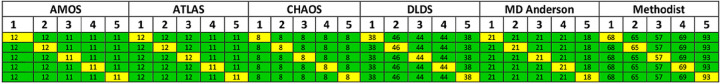
Experiment 1 T1-weighted magnetic resonance imaging dataset split (green = training folds, yellow = validation folds). The numbers of images is indicated in each fold.

We trained and validated SMIT, PocketNet, and nnUNet on only one T1w MRI dataset and repeated this procedure for all six eligible datasets. We performed 5-fold cross-validation to ensure that the model performed inference on every single MR image in the dataset. The splitting is summarized in Fig. 2.

We intended for this experiment to serve as a baseline for comparing each model’s performance on a testing cohort when trained on data drawn from the same source. Because no withheld test set was employed, we treated each model’s validation set as its test set.

In analyzing the results of this experiment, we combined the validation predictions of all five folds for each dataset together. Doing so allowed us to compare the performance of the architectures using predictions for all MR images in each dataset.

### Experiment 2: Leave-One-Dataset-Out Cross-Validation

To follow up on our first experiment, we trained and validated a total of six models on all curated T1w MR images, but with each of the datasets withheld for testing.

Just as our first experiment would demonstrate how each model would perform when tested on data drawn from the same dataset as the data it was trained on, our second experiment would allow us to observe how a model trained on a collection of data from different sources would perform on an unseen cohort of data from a different source.

**Figure 3.**
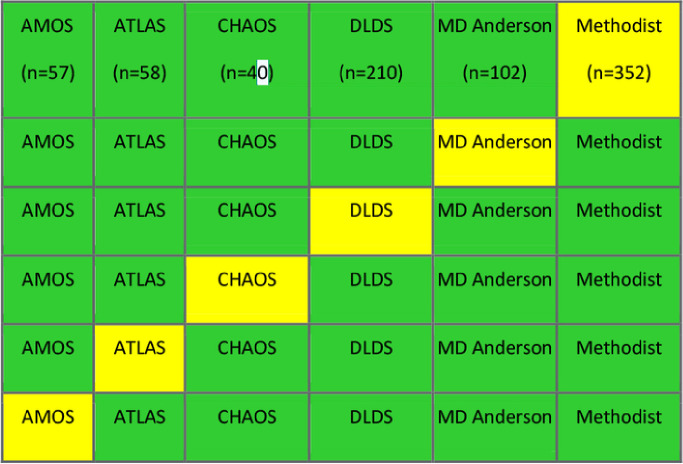
Experiment 2 T1-weighted magnetic resonance imaging dataset split (green = training/validation dataset, yellow = withheld set)

The splitting scheme for this experiment is summarized in Fig. 3. Our working hypothesis was that the testing performance on the withheld dataset would match or exceed the corresponding results from experiment 1 only if the model was trained on images of similar quality or contrast protocol type to those of the dataset that was withheld.

### Quantitative Analysis

For all our experiments, we used three metrics to analyze model performance: DSC, 95th percentile Hausdorff distance, and surface DSC with a tolerance of 2 mm. We chose surface DSC specifically to offset the skew that the large internal volume of the liver can have on the DSC^22^.

### Computational Resources

The SMIT model was coded using MONAI, a PyTorch-based framework for applying deep learning to the field of medical imaging, along with PyTorch itself^23,24^. PocketNet was coded using the Keras library^25^.

To allow for parallel training and testing of both SMIT and nnUNet, we set up pipelines using the scientific pipeline management tool DataJoint^26^. The pipelines also allowed for readily available storage of data and results in tables on databases that are themselves hosted on a MySQL server.

All models were trained on a Kubernetes cluster with 27 nodes, 17 of which had 8 Nvidia A100 graphics processing units, while the other 10 had 8 H100 GPUs.

### Ethics Approval

For this study, retrospective data was collected and analyzed under an approved Institutional Review Board protocol and all consent was waived. All experiments were carried out in accordance with institutional policies.

## Results

### Experiment 1 – One-Source 5-Fold Cross-Validation

#### Quantitative Evaluation

A summary of the quantitative evaluation from SMIT, PocketNet, and nnUNet is provided in Table 3. SMIT achieved mean DSCs ranging from 86.39–96.62%, mean Hausdorff distances ranging from 2.78 mm to 11.98 mm, and mean surface DSCs ranging from 63.33–93.26%. SMIT scored the shortest mean Hausdorff distances of the three models on the MD Anderson and Methodist datasets, achieving 7.80 mm and 6.84 mm, respectively.

PocketNet achieved a similar mean DSC range to that of SMIT, from 86.88–94.71%, but also achieved larger mean Hausdorff distances, ranging from 8.88 mm to 27.81 mm. Its mean surface DSC values, however, never exceeded 90%, with a range of 62.95–84.40%.

nnUNet had the best range of mean DSC values, from 92.34–98.10%, the smallest mean Hausdorff distances, ranging from 2.69 mm to 9.22 mm, and the best surface DSC values, ranging from 82.58–96.39%.

**Figure 4:**
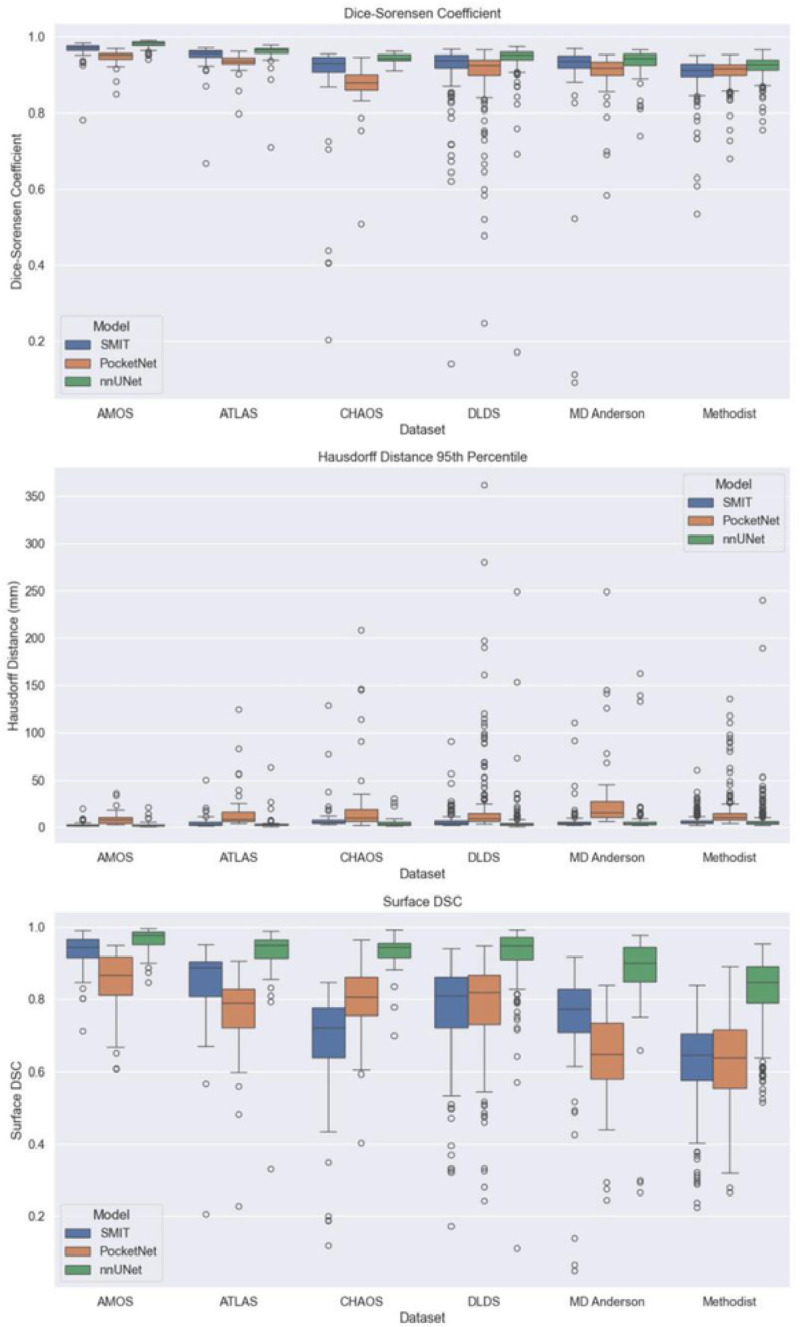
Experiment 1 validation performance metrics

**Figure 5.**
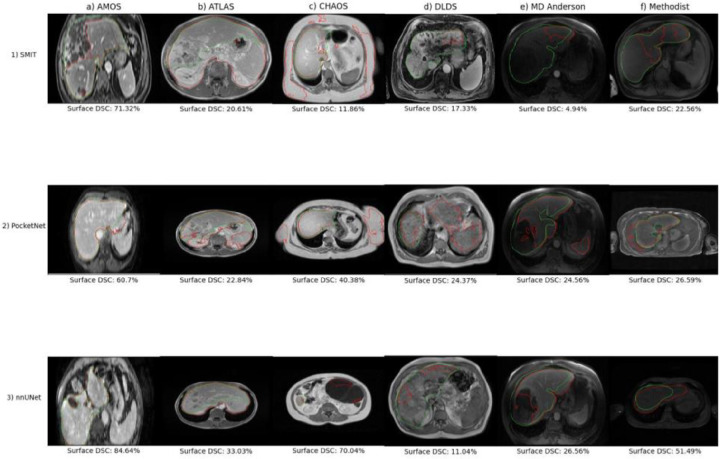
Poorest performing contours as mesasured by the surface Dice-Sorensen coefficient values from experiment 1 models (Ground truth = green, segmentation = red)

Boxplots outlining performance metrics for the models on individual examples are shown in Fig. 4. nnUNet displayed the least variation among the three models, and PocketNet had more variability in its Hausdorff distances than did SMIT and nnUNet. Outliers were caused primarily by under-segmentation of the liver, especially in the presence of motion or noise artifacts and unusual abnormalities in the shape of the liver (Fig. 5 **panels 1a, 1d-f, 3d**), under-segmentation of a tumor or lesion (Fig. 5, **panels 2e, 3e**), and over-segmentation of either the abdominal wall or surrounding organs, such as the spleen and kidney (Fig. 5, **panels b, c, 2a, 2d, 2f, 3f)**.

#### Qualitative Evaluation

**Figure 6.**
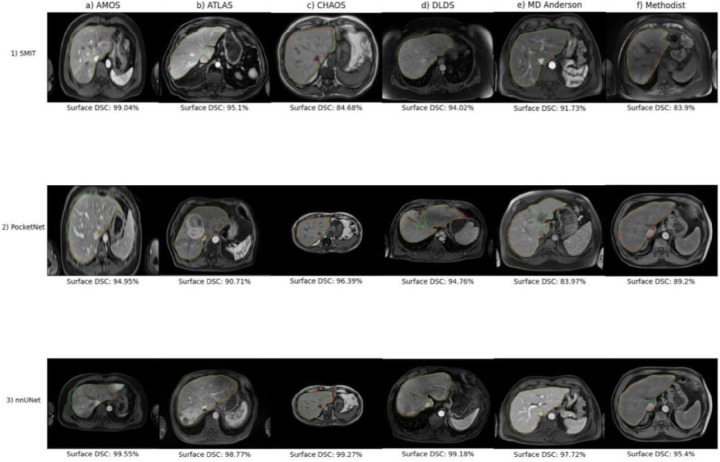
Best performaing contours as mesasured by the surface Dice-Sorensen coefficient values from experiment 1 models (Ground truth = green, segmentation = red)

We plotted the cases with the lowest surface DSC and their respective ground truths for each model trained on each dataset in Fig. 5. Although PocketNet and nnUNet segmented the bulk of the liver when tested on their respective worst cases from the AMOS dataset (Fig. 5, **panels 1a, 2a**), SMIT under-segmented the left lobe due to the presence of a large tumor (Fig. 5, **panel 1a**). All three models performed worst on the same MR image from the ATLAS dataset, which showed severe over-segmentation of the spleen (Fig. 5, **panel b**). It is believed that this common failure was due to intensity homogeneity between the liver and the spleen and the lack of a distinct boundary between the two organs in this MR image. On their worst cases from the CHAOS dataset, SMIT and PocketNet segmented the abdominal wall (Fig. 5, **panels 1–2c**), and nnUNet segmented parts of the heart (Fig. 5, panel 3c). Both SMIT and nnUNet severely under-segmented their respective outlier cases when trained solely on the DLDS (Fig. 5, panel 1d, 3d), and PocketNet segmented parts of the spleen along with the liver (Fig. 5, **panel 2d**). When trained on the MD Anderson dataset, both nnUNet and PocketNet under-segmented the left liver lobe on a portal venous phase MR image from a patient with a large lesion in this lobe (Fig. 5, panel 2–3e), with PocketNet segmenting part of the spleen. SMIT completely under-segmented the entire liver on an arterial phase MR image from this same patient (Fig. 5, **panel 1e**). The model displayed a similar error on its worst outlier from being trained on the Methodist dataset (Fig. 6, **panel 1f**). By contrast, both PocketNet and nnUNet over-segmented the surrounding tissue on their respective outlier cases from this dataset (Fig. 5, **panel 2–3f).**

Contours from each model with the highest surface DSCs and their ground truths are plotted and displayed in Fig. 6. Notable errors were under-segmentation and over-segmentation of the inferior vena cava (Fig. 6, **panels c, 2f),** although this discrepancy could be attributed to interobserver variability across datasets.

### Experiment 2 – Leave-One-Dataset-Out Cross-Validation

#### Quantitative Evaluation

Our hypothesis for experiment 2 was that each model’s performance when tested on a withheld dataset would not exceed the performance of each constituent model from experiment 1. Table 4 outlines the performance metric ranges observed in this experiment.

The mean DSC values for SMIT were lower in experiment 2 than in experiment 1, ranging from 86.23–92.14%. Mean Hausdorff distance values for SMIT were significantly higher, ranging from 7.05 mm to 13.38 mm. Mean surface DSC values for SMIT were lower, ranging from 54.54–81.02%.

Although SMIT recorded a slightly higher DSC when the CHAOS dataset was withheld compared with when SMIT was trained only on this dataset, SMIT also recorded worse mean Hausdorff distances and surface DSC values. SMIT also recorded a slightly lower mean Hausdorff distance when tested on the MD Anderson dataset, but because the mean DSC and mean surface DSC values were lower, there was no clear indication of an improvement in performance.

Like SMIT, PocketNet’s mean DSC values were only slightly different from those observed in experiment 1, ranging from 87.62–93.30%. PocketNet also had an improved mean DSC value when tested on the CHAOS dataset compared with its constituent model in experiment 1. However, in contrast to SMIT, PocketNet also recorded shorter mean Hausdorff distances and higher mean surface DSC values compared with the results of experiment 1, indicating overall better performance on the dataset. We observed a similar, albeit slight, jump in performance on the MD Anderson dataset. Finally, although PocketNet recorded only slightly higher mean DSC values and lower mean Hausdorff distances compared with experiment 1, it also recorded worse mean surface DSC values. Compared with the other two architectures used in experiment 2, PocketNet recorded the largest range of mean Hausdorff distances, from 13.74 mm to 34.07 mm. Mean surface DSC values for PocketNet did not change significantly compared with its constituent results in experiment 1, ranging from 63.94–85.34%.

All mean DSC values for nnUNet were above 90%, ranging from 90.83–96.51%. nnUNet also had the lowest mean Hausdorff distances of the three models, ranging from 4.29 mm to 11.78 mm, and nnUNet was the only architecture of the three to score mean surface DSC values higher than 90%, ranging from 78.45–92.52%. However, nnUNet also achieved a shorter mean Hausdorff distance of 6.69 mm when tested on the MD Anderson dataset compared with when the model was trained only on this dataset in experiment 1, in which its mean Hausdorff distance was 9.22 mm.

Like PocketNet, nnUNet also achieved better performance when tested on the MD Anderson dataset compared with experiment 1, although there was a much clearer improvement in its mean Hausdorff distance compared with its mean DSC and mean surface DSC. nnUNet also achieved a significantly shorter mean Hausdorff distance when trained on the CHAOS dataset than it did in experiment 1, but it also scored a lower mean DSC and mean surface DSC.

**Figure 7:**
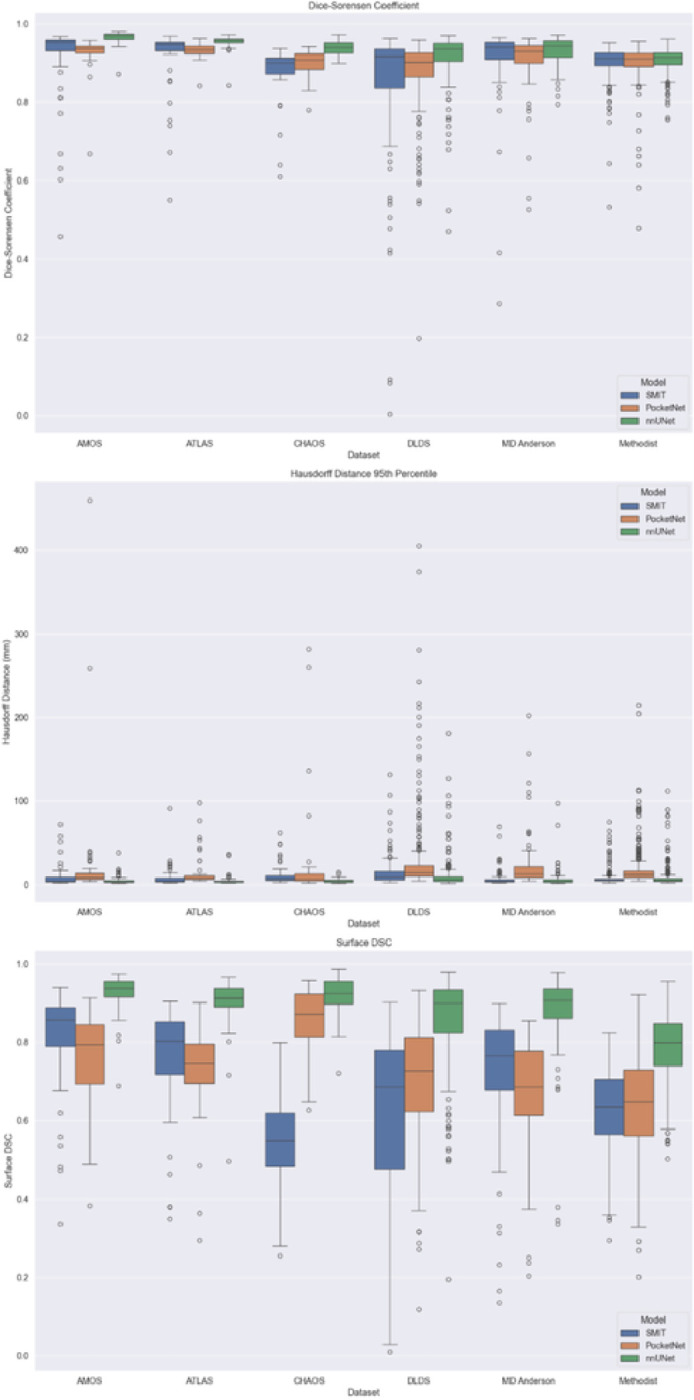
Experiment 2 testing performance boxplots

**Figure 8.**
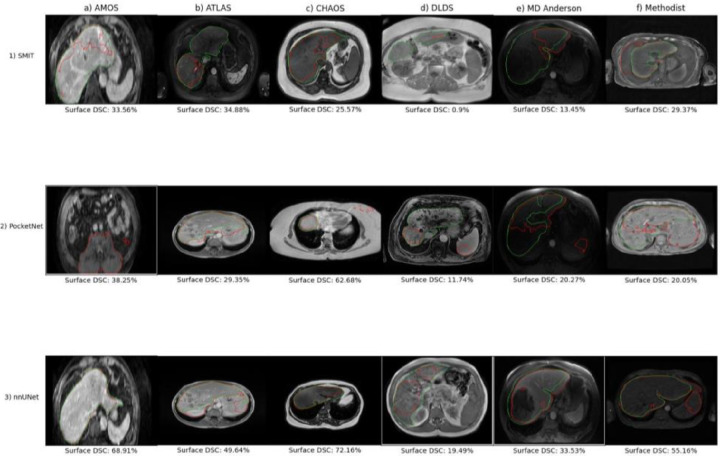
Contours of outlier test cases with low surface Dice-Sorensen Coefficients for experiment 2 models

Boxplots showing the performance distribution are shown in Fig. 7. As was observed in experiment 1, nnUNet displayed the least variation in its performance of the three models across all six withheld datasets and all three performance metrics. PocketNet displayed the most variation of the three models in its Hausdorff distances across all datasets. Of the three performance metrics, surface DSC had the most variability, especially when the models were tested on the DLDS. Outliers were caused primarily by under-segmentation due to factors such as MRI artifacts and abnormalities in liver shape due to the presence of either cirrhosis or lesions (Fig. 8 **panels 1–3d, 1–3e, 1a-b)**, as well as over-segmentation of tissue surrounding the liver when the models were confronted with blurred or otherwise distorted boundaries of the target organ (Fig. 8, **panels 1–3f, 2b-c, 3b)**. These causes were reflected in the larger number of outliers that appeared when the models were tested on the DLDS compared with the other datasets.

#### Qualitative Evaluation

Contours from the testing cases with the lowest surface DSCs are plotted with their ground truths in Fig. 8. One of the most unusual outliers was observed when PocketNet was tested on the AMOS dataset (Fig. 8, **panel 2a)**. Although PocketNet segmented the liver, it also segmented a completely unrelated area in the pelvic region. Equally bizarre was the worst case from SMIT when tested on DLDS (Fig. 8, **panel 1d).** Aside from a small sliver of the right lobe, the model completely failed to segment the organ.

Both SMIT and nnUNet under-segmented their respective worst cases from testing on the AMOS dataset (Fig. 8, **panel 1a, 3a)**, although nnUNet could at least segment the bulk of the liver. SMIT also severely under-segmented its worst case from the MD Anderson dataset, only accounting for segments 2 and 3 (Fig. 8, **panel 1e).** We observed the opposite result in SMIT’s worst case from the ATLAS dataset (Fig. 8, **panel 1b),** in which SMIT segmented only the left liver lobe.

nnUNet and PocketNet over-segmented their respective worst cases from the ATLAS dataset (Fig. 8, **panel 2–3b)**. The MR image on which they performed poorly was scanned in such a setup that at various slices the boundary of the liver was only faintly visible. This was the same MR image that was plotted as the worst case for both models from the ATLAS dataset in experiment 1 (Fig. 5, **panel 2–3b)**, and a comparison of the contour plots suggested that the over-segmentation in experiment 2 was not as severe.

As with its worst case in the AMOS dataset, PocketNet also segmented parts of unrelated tissue in its worst case in the CHAOS dataset (Fig. 8, **panel 2c)**. Except in this case, the over-segmentation was limited to a small portion of the abdominal wall. Both SMIT and nnUNet under-segmented the right liver lobe in their respective outliers from CHAOS (Fig. 8, **panel 1c, 3c).**

The worst DLDS MRI for PocketNet was a portal venous MR image taken from a patient with severe biliary dilatation and atrophy of the right liver lobe (Fig. 8, **panel 2d).** It appeared that due to the dark appearance of the enlarged biliary ducts, the model mistook this region for the boundary of the liver, causing it to severely undersegment the organ while also oversegmenting the spleen. Although nnUNet also undersegmented its worst case from the DLDS dataset, its errors likely occurred due to the presence of image artifacts rather than features specific to the liver itself.

When the two models were tested on their respective worst cases from the MD Anderson dataset, PocketNet counted only the right liver lobe as part of its segmentation (Fig. 8, **panel 2e).** Coincidentally, nnUNet’s outlier was the same MR image that was its outlier when trained on this cohort in experiment 1 (Fig. 8, **panel 3e;** Fig. 6, **panel 3e).** Although nnUNet over-segmented the tissue surrounding the right liver lobe, the model proved more robust to the presence of the large lesion in the left liver lobe, which was contoured along with the surrounding liver tissue.

Finally, all three models over-segmented tissue surrounding the liver when tested on their respective worst cases from the Methodist dataset, with SMIT segmenting the area around the left liver lobe, and both PocketNet and nnUNet segmenting the spleen along with the liver (Fig. 8, **panel 1–3f).**

**Figure 9.**
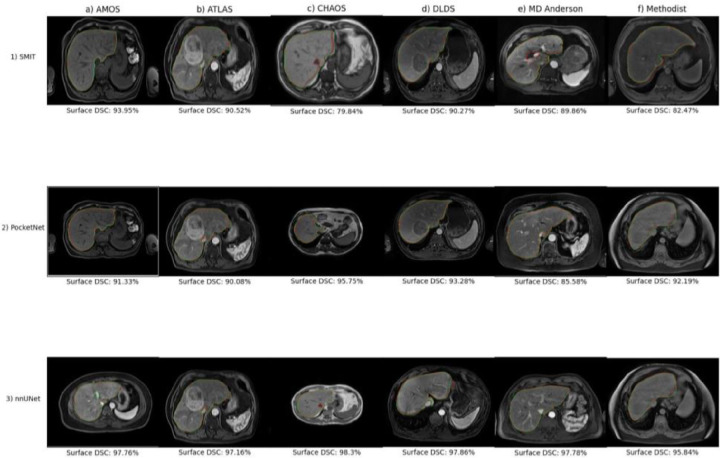
Test cases with the highest surface Dice-Sorensen coefficient values from experiment 2 models

The withheld images for each model that scored the highest surface DSC values are plotted in Fig. 9 **with their respective contours and ground truths.** Both SMIT and PocketNet scored the highest on the same MR images from AMOS and DLDS, and both PocketNet and nnUNet scored the highest on an image from the same patient from the Methodist dataset. All three models scored the highest on the same image from the ATLAS dataset (Fig. 9, **panel b).** The most noticeable discrepancies included over-segmentation around the common hepatic duct (Fig. 9, **panel 1e),** over-segmentation of the middle hepatic vein on the best results from the CHAOS dataset (Fig. 9, **panel 3c),** and under-segmentation of the left portal vein (Fig. 9, **panel 3a).**

## Discussion

Our goal was to use three deep learning architectures and as many T1w MR images as we could gather from multiple institutions to train a robust liver segmentation model that could be applied across MRI vendors and liver disease etiologies. From a supervised learning perspective, such a model would be trained on a sufficiently large and diverse cohort of MR images that encompassed as many etiologies, contrast agent types, and artifacts as could be found. Our experiment 2 models and their results when tested on their respective withheld datasets provided us with an approximation of how each of the three architectures might perform when confronted with a new dataset.

According to the results of our experiments, the best architecture to use for such a model was nnUNet, which scored the highest for all three of our performance metrics in both experiments 1 and 2 and displayed the least variance. Its results in experiment 2 indicated that nnUNet was also the most robust architecture that we used, especially when considering its worst cases (Fig. 9, **panel 3).** Although nnUNet severely under-segmented its worst case from being tested on DLDS, nnUNet was nevertheless able to segment most of the overall liver even when confronted with anomalies such as the enormous lesion that was encountered in its worst case from the MD Anderson dataset.

We also hypothesized that the models in experiment 2 that were each trained with one of the six datasets withheld for testing would not outperform the models from experiment 1 that were trained on only one of these datasets unless their training data contained images of similar quality and sequences. After training on all other datasets, both nnUNet and PocketNet exhibited superior performance across all 3 metrics when tested on the MD Anderson dataset in experiment 2 compared with their performance when trained on only that dataset in experiment 1, and although SMIT showed an improvement in only its mean Hausdorff distances, it did exhibit less variance in both mean DSC values and mean Hausdorff distances, which could constitute an improvement. This boost in performance for all three architectures could be attributed to similar MR images being present in the ensemble of datasets used to train the two models, because both ATLAS and DLDS contain pre-contrast, arterial, and portal venous phase MR images, which make up the entirety of the MD Anderson dataset^6,9^. Additionally, both PocketNet and nnUNet, when tested on the same MR image from the ATLAS dataset, showed qualitative improvement in their contours from experiment 2 (Fig. 9, **panels 2–3b)** compared with those from experiment 1 (Fig. 6, **panels 2–3b)**, which might suggest that the models had been trained on enough MRIs of similar quality and acquisition setting to that particular example. However, although nnUNet achieved slightly similar overall performance on the ATLAS dataset in experiment 2, it did not exceed its experiment 1 results, and PocketNet achieved only a slight improvement in its mean DSC values and mean Hausdorff distances.

We might consider the drop in performance observed across all three models when tested on the DLDS in experiment 2 compared with how they performed when trained only on this dataset in experiment 1 as supporting evidence for our hypothesis, given the large amounts of motion and susceptibility artifacts that are present in the dataset^6^, more so than any other dataset that we used. These artifacts most probably contributed to the SMIT model’s drop in performance, because its surface DSC values were the lowest of all three models when tested on DLDS in experiment 2, and these artifacts also likely worsened the performance of PocketNet and nnUNet. However, another reason for the worsened performance of the models could be the changes in liver shape and appearance caused by cirrhosis, as the worst-case contour plots for PocketNet and nnUNet showed. This would suggest that liver disease etiology was a more significant confounding factor than image quality or contrast type.

Our results present evidence both for and against our experiment 2 hypothesis, and the lack of information regarding contrast types for the AMOS dataset or echo and repetition times for both AMOS and CHAOS are further complications that prevent us from making a proper conclusion on this hypothesis beyond specific datasets.

Our work built upon existing research by training the proven nnUNet on the task of segmenting the liver using 819 T1-weighted MR images gathered mostly from liver cancer patients with different contrast protocols, with performance ranging from comparable to superior when compared against existing models.^2,3,7,8,10,11^ However, unlike Lambert et al’s AHUNets^8^, we did not distinguish between the liver and the tumor and instead counted the latter as part of the former.

Of the six datasets we used in our experiments, only AMOS, ATLAS, CHAOS, and DLDS are publicly available. These MR images account for only 44% of the data that we used to train the model, with most having been curated by MD Anderson and Houston Methodist Hospital. This means that only the results from our experiment 1 models that were trained on these datasets are reproducible. Furthermore, although aggregating multiple datasets did allow us to build a sizable and diverse group of MR images for training, validating, and testing our models, the fact that these datasets were labeled by different individuals introduces the issue of interobserver variability between ground truths, which can easily lead to confusion during the training process. Unfortunately, unless one or more trained radiologists are willing to manually edit over 800 liver contours to ensure uniformity across datasets, there is no easy fix to this limitation.

Work by Isensee et al that compared the rankings of models submitted to a kidney and kidney tumor segmentation challenge indicated that changes to external parameters such as the learning rate, patch sizes, loss functions, and preprocessing schemes had a more significant impact on performance than changes to actual network architecture^3^. Future work might involve additional refinement of the “method configuration,” as Isensee et al collectively referred to these parameters, to determine if performance on liver segmentation is impacted for better or for worse. Additional avenues of exploration include further training of our nnUNet models on any additional T1-weighted liver MRI datasets that have been made public since the start of our research, applying our methodology to T2-weighted MRI datasets, or training combined T1 and T2 nnUNets with our methodology. Aside from obtaining more data, however, future work that might involve refining the data that we have involves image de-noising. Cui et al recently used a 2D convolutional neural network and k-space analysis to reduce and remove motion artifacts from corrupted T2-weighted brain MR images^27^. Given both the prevalence of motion artifacts in DLDS and the fact that, as Macdonald et al noted, such artifacts are not uncommon in a clinical setting^6^, an algorithm that can be applied to remove motion artifacts from liver MR images would expedite the training of robust deep learning segmentation models to assist in preventive surgery.

## Conclusion

We sought to train a robust and generalizable liver T1-weighted MRI segmentation model across different contrast protocols and disease etiologies. Of the architectures we trained using an ensemble of curated data drawn from multiple datasets, we found that models trained using nnUNet were the most robust to changes in image and target organ appearance due to a difference in imaging or health factors. The fact that we observed this trend across all six dataset ensembles we used suggests that any nnUNet model trained on an ensemble of T1-weighted MR images of similar or greater size and diversity will also demonstrate this generalizability.

## Figures and Tables

**Table 1:** Echo time (TE), repetition time (TR), and contrast agents used in magnetic resonance imaging recorded in the publicdatasets

Dataset	TR (ms)	TE (ms)	Contrast agent	Acquisition timing
CHAOS	N/A	N/A	N/A	N/A
DLDS				
In phase	3.84–175	2.46–7.38	Gadobenate dimeglumine (0.1 mL/kg) Gadoxetate disodium (0.05 mL/kg) Rate of infusion 2 mL/s	Arterial phase at 15 seconds; portal venous phase at 70 seconds
Out of phase	3.84–175	1.23–6.15		
Non-contrast	3.46–9.2	1.07–3.13		
Contrast-enhanced	2.83–6.96	1.23–3.27		
AMOS	N/A	N/A	N/A	N/A
ATLAS	3.09–6.78	1.07–4.19	Gadolinium-based contrast	Arterial (early, late) at 12–30 seconds; portal venous phase at 65–70 seconds; delayed at 180–300 seconds

Ms = milliseconds ml = milliliter kg = kilogram

**Table 2:** T1-weighted magnetic resonance imaging data distribution

Dataset	No. patients	No. images	Participants’ findings	Voxel spacing range, mm			No. patients with duplicate images	Cause for duplication	Image distribution
				x	y	z			
CHAOS	20	40	Healthy individuals	0.7–0.8	0.7–0.8	0.7–0.8	20	Dual phase images (each phase = 1 image)	In phase: n = 20; out of phase: n = 20
DLDS	72	210	Cirrhosis	0.6–1.8	0.6–1.8	2.4–10.0	64	Different types of contrast	In phase non-fat saturation: n = 56; late dynamic: n = 2; out of phase: n = 36; precontrast fat suppressed: n = 54; early arterial: n = 1; midarterial: n = 3; portal venous: n = 58
AMOS	57	57	Liver cancer (small sample)	0.6–2.0	0.6–3.0	0.8–3.0	0	N/A	Not provided
ATLAS	58	58	Hepatocellular carcinoma	0.6	0.6	1.4	0	N/A	Fat saturated: n = 58 (precontrast, arterial, portal venous)
Houston Methodist	71	352	Hepatocellular carcinoma	0.6–1.4	0.6–1.4	2.2–4	70	Different scanning protocols	Delayed postcontrast fat suppressed: n = 352
MD Anderson	34	102	Liver cancer	0.6–1.6	0.6–1.6	2.0–3.5	34	Different phases of contrast	Precontrast: n = 34; arterial phase: n = 34;portal venous phase: n = 34
Total	312	819	-	-	-	-	-	-	-

**Table 3:** Experiment 1 validation metric means and standard deviations for SMIT, PocketNet, and nnUNet

Dataset	Dice-Sorenson coefficient		Hausdorff distance 95th percentile		Surface Dice-Sorenson coefficient	
	Mean	Standard deviation	Mean	Standard deviation	Mean	Standard deviation
SMIT						
AMOS	96.62%	2.79%	2.78 mm	2.90 mm	93.26%	5.30%
ATLAS	94.89%	4.16%	5.18 mm	7.27 mm	84.17%	11.35%
CHAOS	86.39%	17.86%	11.98 mm	22.92 mm	65.82%	19.43%
DLDS	92.07%	7.51%	6.48 mm	8.81 mm	77.59%	12.49%
MD Anderson	91.37%	12.45%	7.80 mm	14.58 mm	74.29%	14.77%
Methodist	90.56%	4.26%	6.84 mm	5.72 mm	63.33%	10.46%
PocketNet						
AMOS	94.71%	1.92%	8.88 mm	7.06 mm	84.40%	9.42%
ATLAS	93.29%	2.38%	15.95 mm	20.49 mm	75.93%	10.88%
CHAOS	86.88%	6.92%	27.81 mm	46.54 mm	79.75%	10.84%
DLDS	90.13%	8.45%	22.07 mm	42.12 mm	78.28%	12.37%
MD Anderson	90.75%	5.27%	25.00 mm	32.64 mm	64.46%	11.72%
Methodist	91.12%	2.92%	14.09 mm	15.91 mm	62.95%	12.50%
nnUNet						
AMOS	98.10%	1.06%	2.69 mm	3.55 mm	96.39%	3.23%
ATLAS	95.84%	3.58%	4.42 mm	8.85 mm	92.50%	8.95%
CHAOS	94.34%	1.31%	5.45 mm	6.40 mm	92.85%	5.39%
DLDS	94.23%	5.84%	6.46 mm	20.90 mm	92.51%	8.38%
MD Anderson	93.21%	3.63%	9.22 mm	24.23 mm	87.24%	11.85%
Methodist	92.34%	2.62%	7.79 mm	17.35 mm	82.58%	9.01%

**Table 4:** Experiment 2 validation metric means and standard deviations for SMIT, PocketNet, and nnUNet

Dataset	Dice-Sorenson coefficient		Hausdorff distance 95th percentile		Surface Dice-Sorenson coefficient	
	Mean	Standard deviation	Mean	Standard deviation	Mean	Standard deviation
SMIT						
AMOS	91.32%	10.09%	9.47 mm	13.89 mm	81.02%	12.82%
ATLAS	92.14%	7.54%	8.04 mm	12.89 mm	76.18%	13.17%
CHAOS	87.71%	7.22%	12.62 mm	14.23 mm	54.54%	11.75%
DLDS	86.23%	13.97%	13.38 mm	15.75 mm	62.57%	18.91%
MD Anderson	91.35%	9.17%	7.05 mm	9.81 mm	72.38%	15.46%
Methodist	90.45%	3.86%	7.14 mm	8.54 mm	63.12%	10.02%
PocketNet						
AMOS	92.94%	3.84%	23.72 mm	67.91 mm	75.56%	12.55%
ATLAS	93.30%	1.79%	13.74 mm	18.10 mm	72.92%	10.56%
CHAOS	90.09%	3.22%	25.76 mm	61.84 mm	85.34%	8.69%
DLDS	87.62%	9.18%	34.07 mm	57.55 mm	70.20%	14.51%
MD Anderson	90.96%	7.01%	22.16 mm	30.32 mm	67.89%	12.89%
Methodist	90.23%	4.50%	17.69 mm	22.76 mm	63.94%	12.58%
nnUNet						
AMOS	96.51%	1.58%	5.19 mm	5.87 mm	92.52%	4.84%
ATLAS	95.43%	1.74%	5.11 mm	6.06 mm	89.80%	6.96%
CHAOS	93.80%	1.73%	4.29 mm	2.78 mm	91.56%	5.51%
DLDS	91.73%	6.29%	11.78 mm	20.82 mm	85.32%	12.11%
MD Anderson	93.31%	3.27%	6.69 mm	12.08 mm	87.54%	11.37%
Methodist	90.83%	2.85%	8.51 mm	12.99 mm	78.45%	8.46%

## Data Availability

Of the datasets that we used to train our models, AMOS, ATLAS, CHAOS, and DLDS are publicly available^5,6,9,18^. The MR images from MD Anderson and Houston Methodist Hospital are not publicly available at this time.
